# A Short-Snouted, Middle Triassic Phytosaur and its Implications for the Morphological Evolution and Biogeography of Phytosauria

**DOI:** 10.1038/srep46028

**Published:** 2017-04-10

**Authors:** Michelle R. Stocker, Li-Jun Zhao, Sterling J. Nesbitt, Xiao-Chun Wu, Chun Li

**Affiliations:** 1 Department of Geosciences, 4044 Derring Hall, Virginia Polytechnic Institute and State University, Blacksburg, Virginia, 24061, USA; 2 Zhejiang Museum of Natural History, 6 Westlake Culture Square, Hangzhou, Zhejiang Province 310014, China; 3 Canadian Museum of Nature, P.O. Box 3443, Station “D”, Ottawa, ON K1P 6P4, Canada; 4 Laboratory of Evolutionary Systematics of Vertebrates, Institute of Vertebrate Paleontology and Paleoanthropology, Chinese Academy of Sciences, P.O. Box 643, Beijing 100044, P. R. China

## Abstract

Following the end-Permian extinction, terrestrial vertebrate diversity recovered by the Middle Triassic, and that diversity was now dominated by reptiles. However, those reptilian clades, including archosaurs and their closest relatives, are not commonly found until ~30 million years post-extinction in Late Triassic deposits despite time-calibrated phylogenetic analyses predicting an Early Triassic divergence for those clades. One of these groups from the Late Triassic, Phytosauria, is well known from a near-Pangean distribution, and this easily recognized clade bears an elongated rostrum with posteriorly retracted nares and numerous postcranial synapomorphies that are unique compared with all other contemporary reptiles. Here, we recognize the exquisitely preserved, nearly complete skeleton of *Diandongosuchus fuyuanensis* from the Middle Triassic of China as the oldest and basalmost phytosaur. The Middle Triassic age and lack of the characteristically-elongated rostrum fill a critical morphological and temporal gap in phytosaur evolution, indicating that the characteristic elongated rostrum of phytosaurs appeared subsequent to cranial and postcranial modifications associated with enhanced prey capture, predating that general trend of morphological evolution observed within Crocodyliformes. Additionally, *Diandongosuchus* supports that the clade was present across Pangea, suggesting early ecosystem exploration for Archosauriformes through nearshore environments and leading to ease of dispersal across the Tethys.

The Permian-Triassic mass extinction resulted in a colossal change in global vertebrate community structure^1^,^2^. This largest of the mass extinctions transitioned the existing synapsid-dominated fauna to one dominated by reptiles in terms of abundance, size, and taxonomic diversity^3^,^4^, and allowed the diversification of many major groups present today before leading to the well documented, broadly distributed faunal assemblages of the Late Triassic^5^,^6^. Those Late Triassic vertebrate faunal assemblages are composed of multiple well-known archosauriform clades, many of which have a near global distribution at both low and high latitudes^5^,^7^,^8^. These clades possess distinctive morphologies, but in many cases early-branching members of those clades are either unknown or currently unrecognized because of plesiomorphic character states and extensive ghost lineages. However, progress is being made recognizing early members of both the crocodylian-line archosaurs (i.e. *Xilousuchus*^9^) and avian-line archosaurs (i.e. *Asilisaurus*^10^). Incorporation of those taxa into recent time-calibrated phylogenetic analyses of Archosauriformes^9^,^11^ predicted an Early Triassic divergence for nearly all Triassic archosaurs and their closest relatives, like the proposed sister-taxon, Phytosauria^9^,^12^ (but see ref. 13).

Phytosaurs are well known from a near global distribution in the Late Triassic^5^,^14^,^15^, with the earliest recognized taxa (*Wannia scurriensis, Parasuchus*, ‘*Zanclodon*’ *arenaceus*) known from the latest Carnian or earliest Norian^16^,^17^,^18^,^19^. The only specimen that was potentially older was the now-destroyed *Mesorhinosuchus fraasi*, said to be from the Olenekian of Germany, but this age is controversial^15^,^16^,^17^. By the earliest Norian, phytosaurs had achieved their near global distribution across Pangea (i.e., including current-day India, North America, Morocco, and northern Europe), and all Late Triassic phytosaurs possessed the extremely elongated rostrum and dorsally-facing external nares that sit well posterior on the rostrum. Those iconic features were easily recognized to document the fossil record of this clade. Yet, because the focus was on features clearly unique to phytosaurs this was detrimental to placing the clade in a broader evolutionary framework. A recent surge in species descriptions and subsequent taxonomic revisions^16^,^17^,^18^,^20^,^21^,^22^, especially near the base of Phytosauria, has driven a more detailed examination and atomization of the entire phytosaur skull rather than characterizations of the most prominent features (i.e. the elongation and crests of the rostrum). A major challenge for understanding the evolutionary history of this clade is that analyses of ingroup relationships have focused exclusively on cranial features with broad taxonomic sampling, whereas larger analyses including phytosaurs among other archosauriforms sample both cranial and postcranial characters with a maximum of three phytosaurs to represent ~30 million years of evolution of the group^4^,^9^,^23^. This analytical gap hampers reconstruction of the timing and order of acquisition of the classic phytosaur character states from a plesiomorphic short-snouted archosauriform morphology and inhibits interpretation of ancestral body types at the base of Archosauria.

Using a holistic approach by examining the cranium and postcranium, we targeted the Middle Triassic (Ladinian^24^,^25^) taxon *Diandongosuchus fuyuanensis*^26^ (Figs 1 and 2), recently described as the basalmost poposauroid and well nested within crocodylian-line archosaurs. This taxon lacks a number of suchian character states (e.g., a posteriorly-directed calcaneal tuber, long pelvic elements) that should be present if the taxon does represent a poposauroid. Instead, we detail a number of character states from the cranium and postcranium that are only present in phytosaurs and lead us to reinterpret this taxon not as a poposauroid, but as the sister taxon to all other known phytosaurs. This is a major step in linking the crania-heavy analyses of phytosaur ingroup relationships with postcrania-heavy analyses of archosauriform relationships, and, indeed, fills the temporal gap predicted by phylogenies of Archosauriformes and informs our knowledge of body size and morphological changes during this critical span of phytosaur evolution.

*Institutional Abbreviations* –**UCMP**, University of California Museum of Paleontology, Berkeley, California, USA; **USNM**, Smithsonian National Museum of Natural History, Washington, D.C., USA; **ZMNH**, Zhejiang Museum of Natural History, Hangzhou, China.

## Results

### Revised systematic paleontology

Archosauriformes Gauthier 1986 *sensu*^9^.

Phytosauria Jaeger 1828 *sensu*^17^.

Diandongosuchus fuyuanensis Li *et al*. 2012.

### Holotype

ZMNH M8770, a nearly complete skeleton with most of the caudal vertebrae missing.

### Locality and horizon

West of Huangnihe River, southeast Fuyuan County, Yunnan Province: Zhuganpo Member (Ladinian) of the Falang Formation, late Middle Triassic (Chen, 1985).

### Revised diagnosis

*Diandongosuchus fuyuanensis* differs from all other archosauriforms except members of Phytosauria in the possession of the following combination of character states (using analysis based on that of ref. 9): posterodorsal process of premaxilla strongly sutured to maxilla (4-2; shared with Crocodyliformes); more than six premaxillary teeth (6-3); facial portion of maxilla anterior to anterior edge of antorbital fenestra equal in length or longer than portion posterior to anterior edge of fenestra (14-1); entire anterior margin of scapula straight/convex or partially concave (217-0); anterior portion of coracoid distinctly hooked (226-1); ectepicondylar flange of humerus present (234-0); obturator foramen of the pubis modified into a notch that opens medioventrally (281-2); medial side of distal tarsal 4 with foramen/foramina (352-1); articular surface for the fibula on the calcaneum convex and hemicylindrical shaped (378-1); osteoderms covering the appendages (405-1); retroarticular process of the articular and surangular well ventral to the articulation with the quadrate (414-1); lateral margin of the humerus straight from midshaft to proximal portion (415-1). Additionally, the following character states ambiguously support a sister-taxon relationship between *Diandongosuchus* and Phytosauria: length of anterodorsal process of premaxilla greater than the anteroposterior length of the premaxilla (1-1); squamosal with distinct ridge on dorsal surface along edge of supratemporal fossa (49-1); femoral head orientation anterior (60–90 degrees) (305-0).

*Diandongosuchus fuyuanensis* differs from all other members of Phytosauria in the possession of (*=local autapomorphy): anterodorsal (nasal) process of premaxilla extending posteriorly well posterior external naris*; presence of a fossa expanded in anteroventral corner of external naris*; jugal with pronounced longitudinal ridge on lateral surface and anterior process much broader than the posterior process underlying anterior process of quadratojugal; premaxilla with nine teeth; more than one set of paramedian osteoderms dorsal to the cervical series.

### Reappraisal of *Diandongosuchus fuyuanensis* as a phytosaur

**Figure 1:**
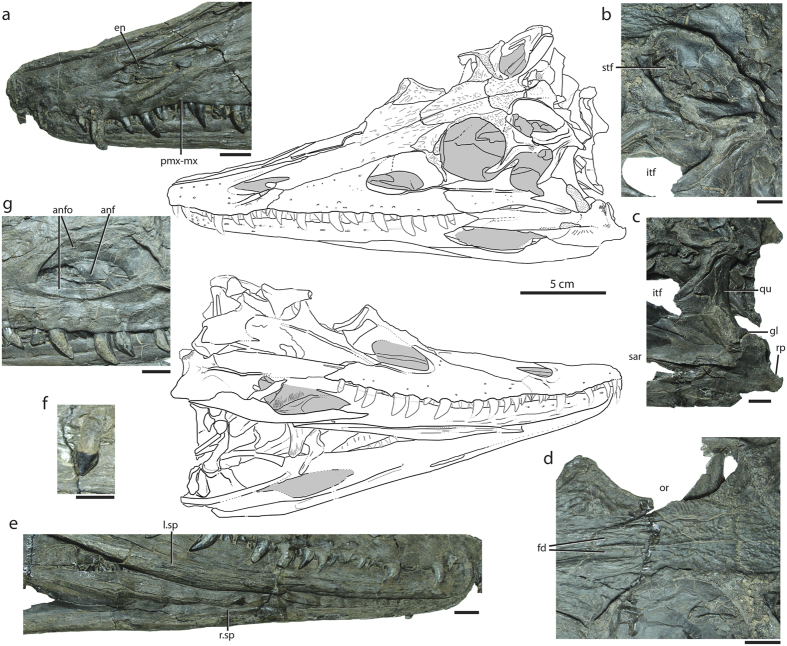
Holotype specimen of *Diandongosuchus fuyuanensis* (ZMNH M8770), showing relevant cranial features shared with Phytosauria. Line drawings of the skull in dorsal (above) and ventral (below) views. (**a**) Anterior portion of rostrum in left lateral view, showing elongated premaxillae and interdigitating premaxilla-maxilla suture; (**b**) left supratemporal fenestra in dorsal view, showing narrow parietal-squamosal bar and fossa in dorsal surface of postorbital-squamosal bar; (**c**) region of left mandibular articulation in left lateral view, showing short retroarticular process well ventral to the distal end of the quadrate; (**d**) skull roof in dorsal view, showing frontal depressions and cranial ornamentation; (**e**) mandibles in right lateral view, showing splenials separated for their length but visible in lateral view along ventral margin of mandibular ramus; (**f**) last maxillary tooth in lateral view, showing spade-shaped morphology; (**g**) region of antorbital fenestra in left lateral view, showing extensive maxillary and lacrimal components to the antorbital fossa. Scale bar for line drawing = 5 cm; scale bars for all other images = 1 cm.

**Figure 2:**
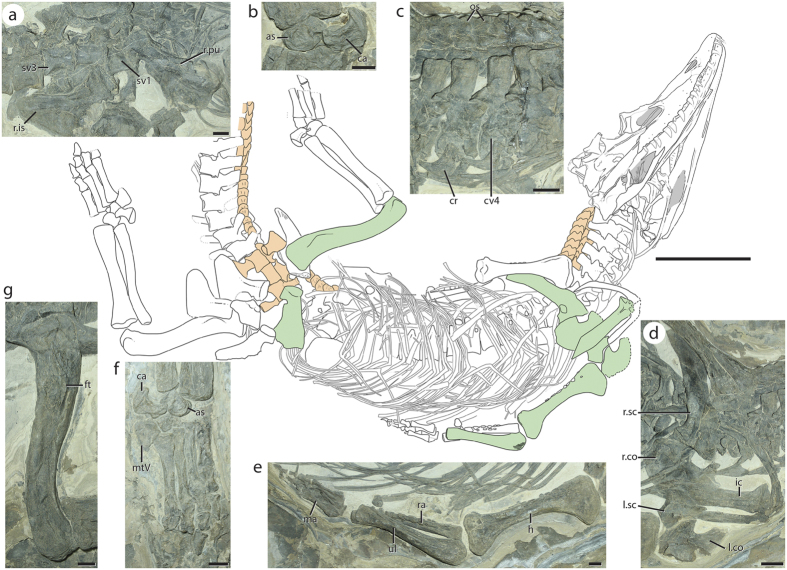
Holotype specimen of *Diandongosuchus fuyuanensis* (ZMNH M8770) in ventral view with relevant postcranial features shared with Phytosauria (in green) and elements with features not found in other phytosaurs (in orange). (**a**) Partially disarticulated pelvis, showing pubic notch and three sacral vertebrae; (**b**) ankle showing a crocodylian-normal pattern; (**c**) cervical region, showing anteroposteriorly short cervical vertebrae and two paramedian columns of osteoderms; (**d**) pectoral region, showing broad interclavicle, backswept scapular blade, and hooked anterior process of the coracoid, (**e**) left forelimb elements, showing straight lateral margin of the humerus and flattened distal end of the ulna; (**f**) left pes, with metatarsal IV as the longest; (**g**) right femur in posterior view, showing folded 4th trochanter. Scale bar for line drawing = 10 cm; scale bars for all other images = 1 cm.

The skull of *Diandongosuchus* possesses many character states only found in phytosaurs, but also lacks some of the hallmark features present in the Late Triassic members of the clade. In *Diandongosuchus*, the position of the nares is retracted from the end of the rostrum (character 139-state 1^9^); this appears to be an intermediate condition between that of archosauriforms (e.g., *Euparkeria*) with nares at the anterior end of the rostrum and derived phytosaurs with the nares near the position of the antorbital fenestrae with the rostrum extended far anteriorly. However, the premaxillae are much shorter than the maxillae in *Diandongosuchus* (10-0^9^), unlike the elongated premaxillae in all other known phytosaurs. The sutural contact between the premaxilla and maxilla is interdigitating (5-2^9^), as in all other phytosaurs, though it does not have the derived ‘zigzag’ morphology (Fig. 1; S Fig. 1). *Diandongosuchus* shares the presence of an antorbital fossa on the posterior process of the maxilla as in Archosauria (137-2^9^) and basal phytosaurs^16^, but here the fossa is nearly continuous along the ventral margin of the antorbital fenestra from the maxilla to the lacrimal. Unlike in all known parasuchids (the node-based group of phytosaurs containing *Wannia scurriensis, Parasuchus hislopi, Mystriosuchus planirostris*, and all descendents of their most recent common ancestor^17^), separate ossifications anterior to the nares and surrounded by the premaxillae (=‘septomaxillae’; see ref. 21 and ref. 9 for further discussion of the homology of these elements) are not externally visible in *Diandongosuchus* (150-0^9^). The jugal bears a strong ridge that trends anteroposteriorly on its lateral surface (75-1^9^) but does not have a row of nodes as is present in *Parasuchus* (44-1^17^), and the anterior process of the element has a small pointed process of the maxilla inserted into the ridge. The jugal is excluded from the antorbital fenestra in *Diandongosuchus* by the lacrimal and maxilla (4-0^17^), but it forms a large portion of the ventral and posteroventral edge of the orbit (5-1^17^). A preorbital depression is present in the prefrontal, as observed in *Parasuchus* and *Mystriosuchus*^17^,^27^. Narrow, anteroposteriorly-oriented depressions in the frontals are located just posterior to the nasal-frontal suture across from the anterior edges of the orbits; these also are present in *Parasuchus* (47-1^17^). A slight dorsally expanded orbital ridge, commonly present in phytosaurs (6-1^17^), is preserved along the left orbit in *Diandongosuchus*. No pineal foramen is present (63-1^9^), consistent with the absence of this feature in other basal phytosaurs such as *Wannia*^18^. The dorsal expression of the postorbital contact with the squamosal (=the postorbital-squamosal bar of phytosaurs) bears a distinct depression similar to that observed in *Parasuchus*^17^, and the dorsal contact of the parietal with the squamosal (=the parietal-squamosal bar of phytosaurs) is mediolaterally narrow, also similar to the morphology observed in *Parasuchus* and other phytosaurs. The quadrate is wide across its ventral condyles in posterior view, a feature also uniquely shared with all other phytosaurs (not currently included in phylogenetic analyses of the clade). The external mandibular fenestra is anteroposteriorly long and dorsoventrally shallow as in phytosaurs (e.g., ref. 22). The long splenials of *Diandongosuchus* have lateral exposure for approximately one-third the length of the ventral edge of the mandible as in other phytosaurs; however, the splenials of *Diandongosuchus* do not meet on the midline to form a symphysis as in all other phytosaurs (160-0^9^;^28^). Additionally, the short posteriorly-directed retroarticular process is well ventral to the articular condyles of the quadrate (S Fig. 2), a feature shared with all other phytosaurs (414-1^9^). The nearly homodont dentition of *Diandongosuchus* consists of recurved, pointed teeth, with the premaxillary and maxillary dentition completely overlapping the dentary teeth labially. All teeth have serrations on their mesial and distal edges; the teeth immediately near the premaxillary-maxillary suture are noticeably smaller than those more anterior or posterior, and the last maxillary tooth is spade-shaped (Fig. 2) in both the left and right maxilla, as is observed in the maxillary dentition of nearly all other phytosaurs^29^.

**Figure 3:**
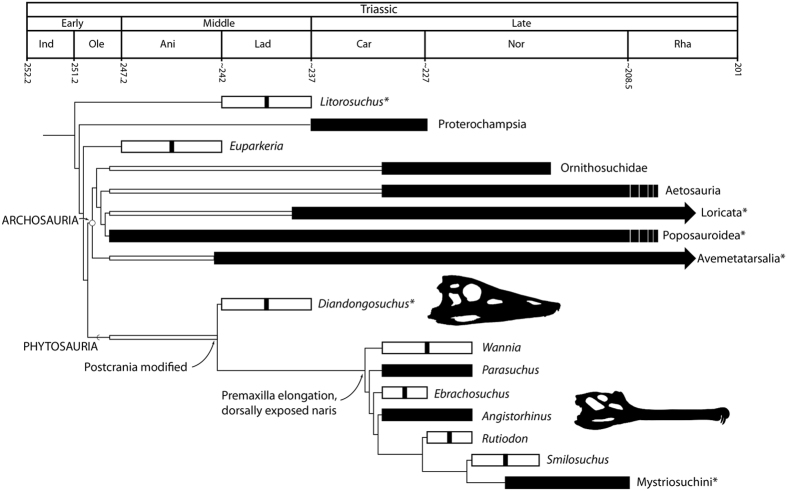
Time calibrated phylogeny of Archosauriformes based on the results of our analyses using the modified matrices of Butler *et al*.^23^ and Kammerer *et al*.^17^. The Ladinian *Diandongosuchus fuyuanensis* is found as the sister taxon to all other phytosaurs, expanding the lineage duration of Phytosauria by 10 million years and showing that premaxillary elongation and dorsal exposure of the nares occurred after postcranial modifications from a plesiomorphic archosauriform morphology. Stars indicate taxa either found in marine sediments or with saltwater tolerant morphological features.

Postcranially, *Diandongosuchus* possesses clear phytosaurian synapomorphies, whether previously documented or newly recognized here. The pectoral girdle of *Diandongosuchus* is distinctly phytosaurian. The scapular blade is backswept from the articular surface for the coracoid and bears a rounded convex process on the anterior portion of the proximal half of the blade as in *Smilosuchus* (USNM 18313). The coracoid has an anterodorsally tapered process with no coracoid foramen (=hooked coracoid, = crescentic coracoid; 226-1^9^). The mediolaterally broad and robust interclavicle has short lateral processes and is flattened dorsoventrally as in other phytosaurs (e.g., USNM 18313). The lateral surface of the humerus has a straight margin from the midshaft to the proximal portion (character 415-1^9^; Fig. 2; S Fig. 3), as in all other known phytosaurs. The pelvic girdle is similar to those of other phytosaurs in that it remains generally plesiomorphic^9^; however, the pubis bears a ventrally open notch for the obturator rather than an enclosed foramen as also observed in *Smilosuchus* (USNM 18313) and possibly unique to phytosaurs. The sigmoidal femur has a 4th trochanter with a concave posterior margin, as also observed in *Smilosuchus* (USNM 18313^9^) and *Machaeroprosopus pristinus* (UCMP 122048: ref. 15: Fig. 4d).

Although *Diandongosuchus* is currently the basalmost phytosaur, it bears a number of features that were not previously predictable for a taxon linking the morphology of typical early archosauriforms (or archosaurs) and phytosaurs. Unlike other phytosaurs, which possess two sacral vertebrae^9^, *Diandongosuchus* possesses three sacral vertebrae in addition to 24 presacral vertebrae. Paramedian osteoderms are present as a left and a right column dorsal to the entire preserved length of the vertebral column as in other phytosaurs and most archosauriforms. However, there are approximately five osteoderms per two vertebrae as in some suchians^9^, whereas other phytosaurs only have one paramedian set of osteoderms per vertebral segment^5^.

### Phylogenetic relationships

We tested the phylogenetic position of *Diandongosuchus fuyuanensis* among Triassic Period Archosauriformes in a modified version (79 taxa and 415 characters) of Nesbitt’s^9^ character-taxon dataset for Archosauriformes, with updates and parameters from a more recent iteration of the original dataset^23^ (see Supplementary Information regarding our additional modifications to the matrix and justifications). Additionally, we included *Diandongosuchus* in the matrix of Ezcurra^13^ to more thoroughly test its phylogenetic relationships among Archosauromorpha and the alternative topology for Phytosauria within Archosauria (see Supplementary Information for parameters used). The systematic relationships of *Diandongosuchus fuyuanensis* among Phytosauria were tested with a modified version of a recent dataset^17^ (based on the original dataset^21^) using their parameters.

We recover *Diandongosuchus* as the sister taxon to all other phytosaurs in both of our analyses of archosauriform relationships and of phytosaurian ingroup relationships (Fig. 3, S Figs 4–6). In the analysis using the Nesbitt^9^ matrix, we recovered 90 most parsimonious trees (MPTs), with tree length of 1340, CI = 0.3627, and RI = 0.7674 (S Fig. 4). Using the Ezcurra^13^ matrix, we recovered 36 MPTs (tree length 2666, CI = 0.2952, and RI = 0.6108), with *Diandongosuchus* recovered as the sister-taxon of all other phytosaurs and Phytosauria as the basalmost clade within Pseudosuchia (S Fig. 5). Using the phytosaur-focused dataset^17^, we recovered 20 MPTs, with tree length of 124, CI = 0.5565, and RI = 0.7669 (S Fig. 6). All other ingroup relationships previously presented^17^ remained the same.

## Discussion

*Diandongosuchus* adds to a growing body of evidence that early archosauriforms and their close relatives possessed a wide range of ecologies^30^,^31^,^32^,^33^,^34^,^35^,^36^. More specifically, *Diandongosuchus* illuminates the order and mode of morphological evolution of Phytosauria through a combination of archosauriform character states and those known for Late Triassic phytosaurs. Several postcranial synapomorphies of Late Triassic phytosaurs are present in *Diandongosuchus*. The uniquely phytosaurian modifications of the pectoral girdle (i.e. large interclavicle, backswept scapular blade, hooked coracoid without a coracoid foramen) remain essentially identical from this point on throughout the existence of the phytosaur lineage, similar to the general postcranial stasis observed in crocodylians^36^,^37^,^38^,^39^. These morphological features were well established prior to the transformation of the skull from a more plesiomorphic archosauriform morphology to the familiar phytosaurian skull.

Some cranial features of Late Triassic phytosaurs also are already present in *Diandongosuchus*, and we hypothesize the potential association of these features with enhanced prey acquisition. This includes the widening of the articulation surface between the skull and mandible through a medially expanded quadrate and articular. This mediolateral expansion and the broadness of the posterior end of the retroarticular process could signal an elongate muscle belly and a larger articulation area (=a bigger mass) for the m. depressor mandibulae and the m. pterygoideus^40^,^41^, providing increased surface for muscle attachment area and possibly increased strength. Furthermore, the premaxilla-maxilla suture is interdigitated, in contrast to the much looser connection between these elements in most other archosauriforms. All these features appeared early in the evolution of Phytosauria prior to the splitting of the *Diandongosuchus* lineage from other phytosaurs, recalling the trend observed in crocodyliform evolution^42^ of later rostrum elongation and dorsal expression of the external nares, as well as increases in body mass and changes in orientation of the temporal musculature, potentially serving to increase bite force and prey capture in a near-shore predator^37^,^43^.

This incorporation of features from the skull and postcranial skeleton has important implications for identifying additional early members of Phytosauria because ingroup relationships currently are based solely on cranial material^16^,^17^,^21^,^27^. Previously, all unambiguous phytosaurian remains were from Upper Triassic sediments^5^,^9^,^15^, despite evidence from time-calibrated phylogenies that the phytosaur clade should extend at least into the late Early Triassic^9^,^11^. Though there have been other specimens that had been thought to be examples of Early or Middle Triassic-aged phytosaurs (e.g., *Mesorhinosuchus fraasi*^44^), those ages were unable to be confirmed and those specimens either lost or not diagnostic to Phytosauria^15^. Our reevaluation of *Diandongosuchus* as a Middle Triassic phytosaur reduces this ghost lineage by ~10 million years (Fig. 3), providing concrete evidence of the early history of this clade, in terms of age as well as in geographic distribution.

Out of all Triassic archosauromorphs, phytosaurs have one of the richest fossil records, preserving one of the widest distributions of reptiles across Pangea. Over the evolutionary history of the clade, phytosaurs achieved a broad biogeographic distribution around the Tethys in humid zones in northern and southern Pangea largely corresponding to the ‘summer-wet-biome’^7^,^45^, but were absent or rare in most of southwest Pangea and southern Gondwana with the exception of a single occurrence from the Santa Maria sequence in present day Brazil^4^,^7^,^46^. By the early part of the Late Triassic, the earliest phytosaurs (e.g., *Parasuchus*) occupied a wide distribution across Pangea^17^, including Germany, Poland, western North America, North Africa, and India, and thus phytosaurs had already achieved a broad biogeographic distribution early in their history. *Diandongosuchus* further widens this early distribution by demonstrating that phytosaurs were present on the far eastern edge of the Pangean supercontinent, indicating additional depositional areas for intense targeted fieldwork to add to the fossil record of these early archosauriforms.

Although widely distributed as a group, individual species of phytosaurs were short lived and were typically replaced by more derived members of the clade in all basins that have a rock record through the end of the Triassic (e.g., Chinle, Dockum, Germanic). As the clade continued to evolve through the Late Triassic those taxa were continually replaced throughout central Pangea rather than forming endemic radiations. This widespread turnover involving redistribution suggests easy dispersal across large portions of the whole of Pangea. It may be tempting to assume that the broad distribution of the group is the result of marine adaptations of some kind as observed in some extant crocodylians^47^,^48^,^49^,^50^. Saltwater tolerance, an extreme marine adaptation, is the result of soft-tissue adaptations including the combination of extremely low skin permeability, the presence of lingual salt glands, and special functions of the kidneys and cloaca^47^, which would be difficult to infer for most fossils. Saltwater tolerance or even possible marine adaptations were hypothesized previously for the phytosaur *Mystriosuchus* from the Upper Triassic of Europe based on both morphological and depositional data^6^,^46^,^51^,^52^,^53^. *Mystriosuchus* bears elongated neural spines, which were cited as supporting a sculling tail for an aquatic ecology^51^. There are no clear morphological features tying *Diandongosuchus* (though fish remains were found as stomach contents in ZMNH M8770^26^) or other phytosaurs to a fully aquatic ecology; however, that paleoecology was implied based on similarities with extant crocodylians^54^. Nevertheless, there are no unambiguous morphological features in the skeletons of extant crocodylians linking them to an aquatic lifestyle. Yet, *Diandongosuchus*, discovered in a marine depositional environment with clearly marine vertebrates and stomach contents that include fish^26^, does provide evidence consistent with a semiaquatic nearshore paleoecology early in the evolution of Phytosauria. Occupation of this nearshore niche could have been a key step in ecosystem exploration leading to the circum-Tethys distribution of phytosaurs, allowing the clade to follow coastlines or navigate open waters and exploit new global opportunities.

## Methods

We incorporated *Diandongosuchus* into the latest iteration of the dataset of Nesbitt^9^ with the rescoring of *Gracilisuchus* and *Turfanosuchus*, the addition of *Yonghesuchus*, and the modifications of character 27 and the addition of character 413 from Butler *et al*.^23^. This resulted in a total of 79 taxa (used *Prestosuchus* combined, *Lewisuchus*/*Pseudoseudolagosuchus* combined, and removed *Archosaurus, Parringtonia*, and *Erpetosuchus*) and 415 characters (character 414 and 415 added here, see below). The rhynchosaur *Mesosuchus browni* was used as the outgroup to root the most parsimonious trees (MPTs). The dataset was analyzed in PAUP*4.0b10^55^ using a heuristic search subjected to 1000 random addition replicates with tree bisection and reconnection branch swapping. Characters 32, 52, 121, 137, 139, 156, 168, 188, 223, 247, 258, 269, 271, 291, 297, 328, 356, 399, and 413 were ordered following Butler *et al*.^23^. Zero-length branches were collapsed if they lacked support under any of the most parsimonious reconstructions. See Supplementary Information for our modifications with explanations to the original scorings for *Diandongosuchus fuyuanensis* from Li *et al*.^26^ into the matrix of Nesbitt^9^, as well as some modifications for other phytosaurs included in the analysis.

To test whether an alternative topology for Phytosauria within Archosauriformes affects the relationships of *Diandongosuchus*, we also incorporated *Diandongosuchus* into the matrix of Ezcurra^13^ (see Supplementary Information for parameters used and results).

Additionally, we incorporated *Diandongosuchus* into the latest iteration of the phytosaur dataset of Stocker^21^ (characters 1–43), with the addition of *Ebrachosuchus neukami* and *Parasuchus angustifrons* and three characters (44–46) and changes in character definitions and taxon scores of Butler *et al*.^16^, as well as the more recent addition of the taxon *Parasuchus hislopi* and characters (47–48) of Kammerer *et al*.^17^. With the addition of *Diandongosuchus*, our final analysis includes 26 taxa and 48 characters. *Euparkeria capensis* was used as the outgroup to root the most parsimonious trees (MPTs). The dataset was analyzed in PAUP*4.0b10^55^ using a heuristic search subjected to 1000 random addition replicates with tree bisection and reconnection branch swapping. Characters 2, 3, and 14 were ordered. Zero-length branches were collapsed if they lacked support under any of the most parsimonious reconstructions. In this analysis we updated character 4 for *Wannia scurriensis* from state (1) to (0) based on the description by Stocker^18^.

## Additional Information

**How to cite this article:** Stocker, M. R. *et al*. A Short-Snouted, Middle Triassic Phytosaur and its Implications for the Morphological Evolution and Biogeography of Phytosauria. *Sci. Rep.* **7**, 46028; doi: 10.1038/srep46028 (2017).

**Publisher's note:** Springer Nature remains neutral with regard to jurisdictional claims in published maps and institutional affiliations.

## Supplementary Material

Supplementary Information
