# Corrigendum: A Short-Snouted, Middle Triassic Phytosaur and its Implications for the Morphological Evolution and Biogeography of Phytosauria

**DOI:** 10.1038/srep46885

**Published:** 2017-10-20

**Authors:** Michelle R. Stocker, Li-Jun Zhao, Sterling J. Nesbitt, Xiao-Chun Wu, Chun Li

Scientific Reports 7: Article number: 46028; 10.1038/srep46028 published online: 04 10 2017; updated: 10 20 2017.

The Supplementary Information file originally published with this Article, contained errors in the Supplementary Figure S6. The taxon ‘*Parasuchus hislopi’* was accidentally inserted twice and ‘*Smilosuchus lithodendrorum’* was inadvertently transposed with *Smilosuchus adamanensis’.* These errors have been corrected in the Supplementary Information that now accompanies the Article.

In addition, three supplementary datasets containing phylogenetic data matrices were omitted from the original version of this Article. These errors have been corrected in the HTML version of the Article; the PDF version was correct at time of publication.

