# Supplementary material for: A Short-Snouted, Middle Triassic Phytosaur and its Implications for the Morphological Evolution and Biogeography of Phytosauria
Source: Sci Rep. 2017 Apr 10;7:46028. doi: 10.1038/srep46028 (PMC5385495; doi:10.1038/srep46028)
Supplement: Supplementary Information [file srep46028-s1.pdf]

Supplementary Information for:

A short-snouted, Middle Triassic phytosaur and its implications for the morphological evolution and biogeography of Phytosauria

Michelle R. Stocker[1*] Li-Jun Zhao[2], Sterling J. Nesbitt[1,] Xiao-Chun Wu[3], and Chun Li[4]

[1]Department of Geosciences, 4044 Derring Hall, Virginia Polytechnic Institute and State University, Blacksburg, Virginia, 24061, USA; stockerm@vt.edu; sjn2104@vt.edu.
[2]Zhejiang Museum of Natural History, 6 Westlake Culture Square, Hangzhou, Zhejiang Province 310014, China;
[3]Canadian Museum of Nature, P.O. Box 3443, Station "D", Ottawa, ON K1P 6P4, Canada;
[4]Laboratory of Evolutionary Systematics of Vertebrates, Institute of Vertebrate Paleontology and Paleoanthropology, Chinese Academy of Sciences, P.O. Box 643, Beijing 100044, P. R. China.

CONTENTS

I. Character Modifications for Phylogenetic Analysis of Archosauriformes including *Diandongosuchus* using matrix of Nesbitt (2011)
II. Parameters used for phylogenetic analysis of *Diandongosuchus* using matrix of Ezcurra (2016)
III. Diagnoses of Phytosauria and Parasuchidae
IV. Support for Phytosauria outside of Archosaurs
V. Supplemental References

Figure S1: Comparisons of the premaxilla-maxilla suture among Archosauriformes,
Figure S2: Comparisons of retroarticular process length and orientation among Archosauriformes.
Figure S3: Comparisons of humeral morphology among Archosauriformes.
Figure S4: Phylogenetic relationships among Archosauriformes using matrix of Nesbitt (2011).
Figure S5: Phylogenetic relationships among Archosauromorpha using matrix of Ezcurra (2016).
Figure S6: Phylogenetic relationships among Phytosauria.

I. Character Modifications for Phylogenetic Analysis of Archosauriformes including *Diandongosuchus* using matrix of Nesbitt (2011)

4. Premaxilla, posterodorsal process (=maxillary process, =subnarial process): (0) fits between the nasal and the maxilla or lies on the anterodorsal surface of the maxilla; (1) overlaps anterodorsal surface of nasal; (2) sutured to maxilla; (3) fits into slot of the nasal. ([1]:Fig. 14) (Fig. S1) (modified from[2-7]).

This character was slightly modified from the original version from Nesbitt[1]. Character state (2) 'vertical, strongly suture to the maxilla' was changed to 'sutured to maxilla' to remove the orientation part of the state. Currently, this state still represents the character state present in crocodyliforms, but also is present in *Diandongosuchus* and all other phytosaurs.

6. Premaxillary teeth, number: (0) 3; (1) 4; (2) 5; (3) 6+; (4) 0.

We changed the formulation back to that of Nesbitt[1]. *Diandongosuchus* and all phytosaurs are scored as character state (3).

22. This character was scored as (0) by Li et al. [8], but should be (?) because the interdental plates are not visible in the specimen.

27. Maxilla, portion of the posterior process that lies ventral to the anterior three-quarters of the antorbital fenestra, not including the posterior end of the maxilla (whether or not it lies below the fenestra): tapers posteriorly (0), is approximately constant in dorsoventral depth (1), or becomes dorsoventrally deeper posteriorly (2). (modification from [9])

This character was modified from state (1) by Li et al. [8] to state (2) based on the modification of Butler et al. [9] and because the posterior process of the maxilla clearly becomes deeper posteriorly.

41. This character was scored as (0) by Li et al.[8], but it cannot be seen so it was changed to (?).

43. This character was scored as state (0) by Li et al.[8], but was changed to state (1) based on a closer examination.

49. We rescored *Parasuchus hislopi* to state (1). There is a slightly developed ridge on the dorsal margin of the squamosal in *Diandongosuchus* and other phytosaurs, but it is not a distinct overhanging ridge as in *Postosuchus kirkpatricki* and crocodylomorphs.

55. This character was scored as state (1) by Li et al. [8], but there is no fossa on the lateral surface of the squamosal as in rauisuchids. Therefore we changed the state to (0).

72. This character was scored as state (0) by Li et al. [8], but the jugal extends far posteriorly, so that the jugal reaches posterior to the infratemporal fenestra. Therefore we score it as state (1).

88. This character was scored as unknown (?) by Li et al.[8]. We rescored this character as state (1) because the ectopterygoid forms all of the lateral pterygoid flange.

127. This character was scored as unknown (?) by Li et al.[8]. We did not observe any ridges on the anterolateral surfaces of the supraoccipital, so we scored the character as state (0).

144. This character was scored as (1) by Li et al.[8], but after a reexamination, we are confident that there is no fossa present anterior to the supratemporal fenestra. Therefore we scored this character as (0).

165. This character was scored as unknown (?) by Li et al.[8]. We scored this character as state (0) because we did not see a foramen on the medial surface of the splenial.

182. This character was scored as (0) by Li et al.[8], but we were not able to see the feature in anterior view. Therefore we scored the character as unknown (?).

183. This character was scored as (1) by Li et al.[8], but we could not make a precise measurement. Therefore, we scored this character as unknown (?).

185. This character was scored as (0) by Li et al.[8], but we changed it to unknown (?) because this part of the cervical vertebrae cannot be seen.

191. This character was scored as state (1) by Li et al.[8], but the compression of the specimen makes this character very difficult to score. It appears that lateral expansions are present at the distal ends of the neural spines of the cervical vertebrae, but we cannot say so confidently. Therefore we scored this character as unknown (?).

210. This character was scored as unknown (?) by Li et al.[8], but we changed it to state (0) because the small processes on the middle caudal vertebrae appears to be absent.

217. We rescored *Smilosuchus gregorii* and *Machaeroprosopus pristinus* as (0) following the scores for *Parasuchus hislopi* and *Diandongosuchus*.

220. This character was scored as (1) by Li et al.[8]. Here we interpret the acromion process of the scapula of *Diandongosuchus* as flat and in the same plane as the ventral edge of the scapula. The state for *Diandongosuchus* is identical to that of phytosaurs. Therefore, we scored this character as state (0).

223. This character was scored as unknown (?) by Li et al.[8], but we changed it to state (0), short postglenoid process.

226. This character was scored as state (0) by Li et al.[8]. Here we changed the score to state (1). *Diandongosuchus*, like all other phytosaurs, has a distinctly hooked anterior margin of the coracoid. This was reconstructed incorrectly by Li et al.[8] when it appeared that the scapula covered the coracoid notch and formed a coracoid foramen; reinterpretation of the articular surfaces puts the scapula more posterior on the coracoid and reveals the distinct anterior hook of the coracoid. Additionally, *Diandongosuchus* and other phytosaurs also lack a coracoid foramen.

228. This character was scored as unknown (?) by Li et al.[8]. The posterodorsal edge of the coracoid lacks a distinct groove so it was changed to state (0).

237. This character was scored as unknown (?) by Li et al.[8], but we changed it to state (0). The lateral surface of the right ulna is exposed in *Diandongosuchus*, and it appears that the surface is

rounded without a distinct lateral tuber as in taxa scored as state (0). The morphology of the ulna is nearly identical to that of other phytosaurs (e.g., *Smilosuchus gregorii*, USNM 18313; [1]:figure 31E).

238. This character was scored as state (1) by Li et al.[8], but we changed it to state (0). The lateral surface of the right ulna is exposed in *Diandongosuchus* and the distal surface is rounded similar to that of *Smilosuchus gregorii* (USNM 18313; [1]:figure 30A) and lacks the distinct squared-off morphology of loricatans.

239. This character was scored as unknown (?) by Li et al.[8]. Here, we scored the character as state (0) because the distal end of the ulna is compressed like that of other phytosaurs and stem archosaurs.

242. This character was scored as state (0) by Li et al.[8], but is rescored as unknown (?) because the proximal carpals are not preserved.

243. This character was scored as state (0) by Li et al.[8], but is rescored as unknown (?) because the proximal carpals are not preserved.

246. This character was scored as unknown (?) by Li et al.[8]. Here, we rescored the character as state (0). The proximal ends of metacarpals I-III of the left manus of *Diandongosuchus* remain in articulation and have the plesiomorphic character state of having a simple overlap with one another.

247. This character was scored as unknown (?) by Li et al.[8]. We took measurements of the forelimb elements and concluded that the manus accounts for less than 0.3 of the total length of the humerus + radius. Hence, we scored this character as state (0).

250. This character was scored as state (0) by Li et al.[8], but we now think that this character cannot be scored because of poor preservation of the manus. Therefore, we scored this character as unknown (?).

256. This character was scored as state (0) by Li et al.[8], but we now think that this character cannot be scored because of poor preservation. Therefore, we scored this character as unknown (?).

257. This character was scored as state (1) by Li et al.[8]. Here we revised this score as state (0) because the one manual ungual that is preserved in *Diandongosuchus*, although it has a sharp tip, is rather short and blunt, and it is not recurved like that of theropod dinosaurs.

259. This character was scored as state (0) by Li et al.[8], but we now think that this character cannot be scored because of poor preservation. Therefore, we scored this character as unknown (?).

260. This character was scored as state (1) by Li et al.[8], but we now think that this character cannot be scored because of poor preservation. Therefore, we scored this character as unknown (?).

261. This character was scored as state (0) by Li et al.[8], but we now think that this character cannot be scored because of poor preservation. Therefore, we scored this character as unknown (?).

269. This character was scored as unknown (?) by Li et al.[8]. The preacetabular process of the ilium is visible in medial view, and it is clear that the preacetabular process is present and short. Therefore, we scored the character as state (0).

275. This character was scored as state (0) by Li et al.[8]. However, the dorsal margin of the ilium is covered by the femur. Therefore, we scored this character as unknown (?).

281. MODIFIED. Pubis, obturator foramen: (0) small; (1) enlarged; (2) modified into a notch that opens medioventrally (following [8]). The pubis of *Diandongosuchus* lacks an obturator foramen. Instead, *Diandongosuchus* has an open notch in the pubis (Fig. 2a) that is likely homologous to the obturator foramen. Therefore, we have revised character 281 by adding state (2), obturator foramen modified into a notch that opens medioventrally. A similar notch is also present in *Smilosuchus gregorii* (USNM 18313), and this character was scored as small (0) for *Parasuchus* based on the description by Chatterjee[10].

283. This character was scored as state (1) by Li et al.[8]. However, The pubic shaft of *Diandongosuchus* is similar throughout its length and does not expand posteriorly into a boot at its distolateral margin as in *Arizonasaurus babbitti* (MSM 4590) and *Postosuchus alisonae* (UNC 15575). Therefore, we scored this character as state (0).

284. This character was scored as state (0) by Li et al.[8]. But, because character 283 is now scored as state (0), character 284 is scored as inapplicable (-).

285. This character was scored as state (1) by Li et al.[8]. But, because character 283 is now scored as state (0), character 285 is scored as inapplicable (-).

286. This character was scored as state (1) by Li et al.[8]. However, this portion of the articular surfaces of the pubis are hidden. Therefore, we score this character as unknown (?).

287. This character was scored as state (1) by Li et al.[8]. Although the pelvis of *Diandongosuchus* is disarticulated, the anteroventral edge of the right ischium indicates that the contact between the ischium and pubis extended ventrally like that of *Euparkeria capensis*. Here, we rescored the character as state (0).

288. This character was scored as state (1) by Li et al.[8]. The proximal articulation surface between the pubes is not relatively thickened relative to the rest of the pubis in *Diandongosuchus* in comparison with poposauroids (e.g., *Shuvosaurus inexpectatus*). Therefore, we scored this character as state (0).

291. This character was scored as state (1) by Li et al.[8]. The ischia of *Diandongosuchus* meet only on the ventromedial edge and do not have extensive contact like those of paracrocodylomorphs[1] and, hence, we scored this character as state (0).

302. This character was scored as state (0) by Li et al.[8]. The femora are too crushed to see the tubera, so we scored this character as unknown (?).

303. This character was scored as state (0) by Li et al.[8]. The femora are too flattened, so we scored this character as unknown (?).

307. This character was scored as state (0) by Li et al.[8]. We scored the character as unknown (?) because the femora are only visible in posterior/ventral view.

308. This character was scored as state (0) by Li et al.[8]. We scored the character as unknown (?) because the femora are only visible in posterior/ventral view.

310. This character was scored as state (0) by Li et al.[8]. We scored the character as unknown (?) because the femora are only visible in posterior/ventral view.

311. This character was scored as state (0) by Li et al.[8]. We scored the character as unknown (?) because the femora are only visible in posterior/ventral view.

312. This character was scored as state (0) by Li et al.[8]. We scored the character as unknown (?) because the femora are only visible in posterior/ventral view.

319. This character was scored as state (0) by Li et al.[8]. We scored the character as unknown (?) because the femora are only visible in posterior/ventral view.

320. This character was scored as state (0) by Li et al.[8]. We scored the character as unknown (?) because the femora are only visible in posterior/ventral view.

321. This character was scored as state (0) by Li et al.[8]. We scored the character as unknown (?) because the femora are flattened and only visible in posterior/ventral view.

322. This character was scored as state (0) by Li et al.[8]. We scored the character as unknown (?) because the femora are flattened and only visible in posterior/ventral view.

323. This character was scored as state (1) by Li et al.[8]. This character can only be scored from a cross section of the femur; the femora of *Diandongosuchus* are complete and in articulation. Therefore, we scored the character as unknown (?).

324. This character was scored as state (0) by Li et al.[8]. We scored the character as unknown (?) because the femora are flattened and only visible in posterior/ventral view.

325. This character was scored as state (0) by Li et al.[8]. We scored the character as unknown (?) because the femora are flattened and only visible in posterior/ventral view.

326. This character was scored as state (0) by Li et al.[8]. We scored the character as unknown (?) because the femora are flattened and only visible in posterior/ventral view.

328. This character was scored as state (0) by Li et al.[8]. This character is scored as unknown (?) because the tibiae are crushed and only visible in posterior view.

330. This character was scored as unknown (?) by Li et al.[8]. The lateral condyles are visible in *Diandongosuchus*, and there is left-right symmetry of a depression in the correct position. Therefore, we scored this character as state (1).

331. This character was scored as unknown (?) by Li et al.[8]. The two proximal condyles of the left tibia of *Diandongosuchus* are well separated, so we scored this character as state (0).

339. This character was scored as unknown (?) by Li et al.[8]. *Diandongosuchus* has a relatively small M. iliofibularis attachment location and is scored as (0) here.

345. This character was scored as unknown (?) by Li et al.[8]. The distal end of the left fibula of *Diandongosuchus* is asymmetrical like that of most pseudosuchians and is scored as (0).

352. This character was scored as state (0) by Li et al.[8]. The medial side of distal tarsal 4 cannot be seen so we scored this character as unknown (?).

354. This character was scored as state (0) by Li et al.[8]. The tibial facet cannot be seen because the ankle is in articulation so we scored this character as unknown (?).

355. This character was scored as state (0) by Li et al.[8]. The tibial facet cannot be seen because the ankle is in articulation so we scored this character as unknown (?).

358. This character was scored as state (0) by Li et al.[8]. The preservations is poor here, so we scored this character as unknown (?).

364. This character was scored as state (0) by Li et al.[8]. The tibial articular surface of the astragalus is not visible, so we scored this character as unknown (?).

365. This character was scored as state (1) by Li et al.[8]. The proximal surface of the astragalus is not visible, so we scored this character as unknown (?).

367. This character was scored as unknown (?) by Li et al.[8]. The peg is clearly visible and indicates a continuous articular surface. Therefore, we scored this character as state (0).

372. This character was scored as state (1) by Li et al.[8]. The right calcaneum of *Diandongosuchus* bears articular facets for the fibula (=roller surface) and the astragalus (slightly concave surface), and these two surfaces are connected like those of other phytosaurs (USNM 18313) and *Euparkeria capensis*[11]. The "peg" of the right astragalus (misidentified by Li et al.[8]) is short and indicates that the articulation of the astragalus and calcaneum would be like that of other phytosaurs (USNM 18313) and *Euparkeria capensis*[11]. Therefore, we rescored this character as state (0).

375. This character was scored as unknown (?) by Li et al.[8]. There is no median depression visible on the calcaneum. Therefore, we scored this character as state (0).

376. This character was scored as state (2) by Li et al.[8]. The calcaneal tuber is not just short of twice the mediolateral width of the fibular facet; it is more likely about as tall as broad. Therefore, we rescored this character as state (1).

377. This character was scored as state (2) by Li et al.[8]. The orientation of the calcaneum with respect to the astragalus of *Diandongosuchus* cannot be determined because the ankle region is slightly disarticulated and compressed. Therefore, we rescored this character as unknown (?).

379. This character was scored as unknown (?) by Li et al.[8]. The calcaneum of *Diandongosuchus* retains the short processes and is rescored as state (0).

397. We rescored *Parasuchus* from state (?) to state (0) based on photos of metatarsal V. No concave gap is present, contra what was shown in figures by Chatterjee[10].

400. We rescored *Parasuchus* from state (1) to state (2) in agreement with the morphology in *Smilosuchus* and *Machaeroprosopus*, as well as in *Diandongosuchus*.

411. This character was scored as state (0) by Li et al.[8]. The trunk vertebrae and associated osteoderms are covered by gastralia and stomach contents in *Diandongosuchus*. Therefore, we scored this character as unknown (?).

413. ADDED FROM BUTLER ET AL.[9]. Maxilla with triangular posterodorsal process possessing clear dorsal apex and formed by discrete expansion of posterior end of horizontal process of maxilla: absent (0); present, but weakly developed, extends dorsal to the dorsal

margin of the horizontal process by a distance that is less than the height of the horizontal process of the maxilla immediately anterior to the posterodorsal process (1); present and strongly developed, extends dorsal to the dorsal margin of the horizontal process by a distance that is almost equivalent to the height of the horizontal process of the maxilla immediately anterior to the posterodorsal process (2). This character is treated as additive/ordered.

414. NEW. Articular and surangular, retroarticular process in lateral view, posterior termination: (0) absent or at a similar horizontal level as the articulation with quadrate; (1) well ventral to the articulation with the quadrate. (Fig. S2)

In taxa such as *Euparkeria* (SAM K 5867; Fig. 2a) and *Postosuchus* (TTU-P 9000) the retroarticular process extends posteriorly from the glenoid either with no dorsally-facing concavity or with a dorsally-facing concavity that is in the same horizontal plane as the glenoid (state 0). *Diandongosuchus* (Fig. 2c) and other phytosaurs are scored as state (1), where the retroarticular process dips ventrally posterior to the glenoid to create a posteriorly-facing concavity that is well ventral to the glenoid articulation.

415. NEW. Humerus, lateral margin of the midshaft to the proximal portion: (0) concave; (1) straight. (Fig. S3)

The proximal half of the lateral margin of the humerus is distinctly concave in *Batrachotomus* (SMNS 80275; Fig. 3a), with the proximal quarter nearest the deltopectoral crest angled outward (state 0). *Diandongosuchus* (Fig. 3c) and phytosaurs are scored as state (1), where the proximal quarter of the lateral margin does not expand outward but keeps the proximal half of the lateral margin in a straight plane.

II. Parameters used for phylogenetic analysis of *Diandongosuchus* using matrix of Ezcurra (2016)

Ordered characters: 1 2 7 10 17 19 20 21 28 29 36 40 42 50 54 66 71 75 76 122 127 146 153 156 157 171 176 177 187 202 221 227 263 266 279 283 324 327 331 337 345 351 352 354 361 365 370 377 379 398 410 424 430 435 446 448 454 458 460 463 472 478 482 483 489 490.

Deleted taxa: *Kadimakara australiensis* holotype, *Chasmatosuchus magnus*, "*Gamosaurus lozovskii*", *Garjainia madiba* holotype, *Uralosaurus magnus* holotype, *Eorasaurus olsoni*, *Prolacertoides jimusarensis*, *Archosaurus rossicus*, *Proterosuchus fergusi* holotype, "*Ankistrodon indicus*", "*Exilisuchus tubercularis*", "*Blomosuchus georgii*", *Vonhuenia friedrichi*, *Chasmatosuchus rossicus*, *Chasmatosuchus magnus* combined (i.e., *Chasmatosuchus magnus* + "*Gamosaurus lozovskii*"), "*Chasmatosuchus*" *vjushkovi*, Long Reef proterosuchid, *Shansisuchus kuyeheensis*, "*Dongusia colorata*", *Uralosaurus magnus* combined, and "*Chasmatosaurus ultimus*", *Asperoris mnyama*, and *Kalisuchus rewanensis*.

Our final matrix includes 600 characters and 80 taxa. The parsimony analysis was run mimicking the parameters used by Ezcurra[12], including ACCTRAN, the setting "collapse branches if maximum length is zero", and multiple states interpreted as uncertainty. The heuristic search used random addition (10 replicates) and TBR branch swapping. This analysis resulted in 36 MPTs of tree length 2666, CI=0.2952, and RI=0.6108. Here, the strict consensus found *Diandongosuchus* as the basal-most known phytosaur, with Phytosauria as the earliest branching clade within Pseudosuchia (Fig. S5).

III. Diagnoses of Phytosauria and Parasuchidae

Phytosauria (stem): *Rutiodon carolinensis*, and all taxa more closely related to it than *Aetosaurus ferratus*, *Rauisuchus tiradentes*, *Prestosuchus chiniquensis*, *Ornithosuchus woodwardi* or *Crocodylus niloticus* sensu Stocker and Butler[13].

Diandongosuchus and all members of Phytosauria share the following unambiguous cranial and postcranial synapomorphies: posterodorsal process of premaxilla strongly sutured to maxilla (4-2; shared with Crocodyliformes); more than six premaxillary teeth (6-3); facial portion of maxilla anterior to anterior edge of antorbital fenestra equal in length or longer than portion posterior to anterior edge of fenestra (14-1); entire anterior margin of scapula straight/convex or partially concave (217-0); anterior portion of coracoid distinctly hooked (226-1); ectepicondylar flange of humerus present (234-0); obturator foramen of the pubis modified into a notch that opens medioventrally (281-2); medial side of distal tarsal 4 with foramen/foramina (352-1); articular surface for the fibula on the calcaneum convex and hemicylindrical shaped (378-1); osteoderms covering the appendages (405-1); retroarticular process of the articular and surangular well ventral to the articulation with the quadrate (414-1); lateral margin of the humerus straight from midshaft to proximal portion (415-1). Additionally, the following character states ambiguously support a sister-taxon relationship between *Diandongosuchus* and Phytosauria: length of anterodorsal process of premaxilla greater than the anteroposterior length of the premaxilla (1-1); squamosal with distinct ridge on dorsal surface along edge of supratemporal fossa (49-1); femoral head orientation anterior (60-90 degrees) (305-0).

Parasuchidae *sensu* Kammerer et al.[14] is supported by the following unambiguous synapomorphies in our archosauriform phylogeny: premaxilla longer than maxilla (10-1);

posterior edge of maxillary teeth convex (15-1); anterior extent of maxilla anterior to nasals (19-1); quadratojugal subtriangular (46-1); dorsal head of quadrate with a sutural contact with the paroccipital process of the opisthotic (77-1); external nares nonterminal, posterior rim of nares anterior to anterior rim of antorbital fenestra (139-1); external nares directed dorsally (140-1); presence of separate ossification anterior to the nasals surrounded by the premaxilla (150-1); dentary-splenial mandibular symphysis present along one-third of lower jaw (160-1); dentition markedly heterodont (167-1); attachment site for the M. iliofibularis on the fibula knob shaped and robust (339-1). Additionally, the following ambiguous characters states may support Parasuchidae as well: antorbital fenestra restricted to the lacrimal and dorsal process of the maxilla (137-1); accessory laminar process of middle caudal vertebrae present on anterior face of neural spine (210-1); ratio of longest metacarpal to longest metatarsal greater than 0.5 (245-0).

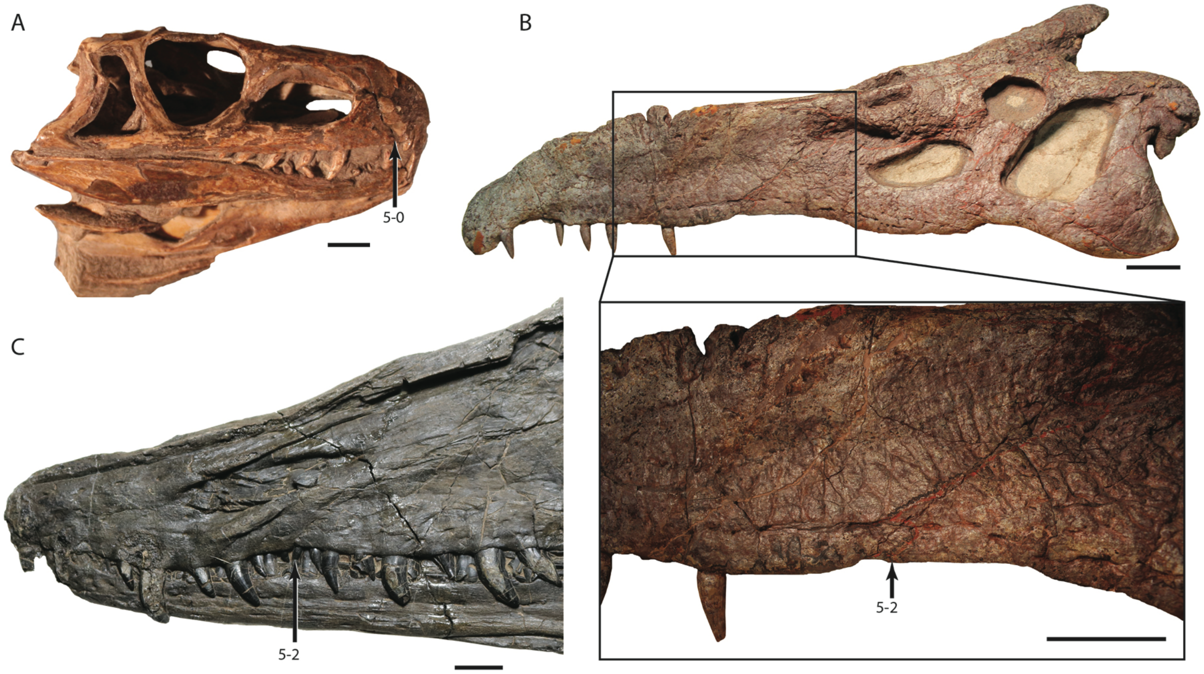

Figure S1. Comparisons of the premaxilla-maxilla suture among Archosauriformes, A. Holotype skull of *Euparkeria capensis* (SAM K 5867) in right lateral view; B. Skull of *Nicrosaurus kapffi* (SMNS 5726) in left lateral view, with close up of premaxilla-maxilla suture highlighted in red; C. Anterior half of holotype skull of *Diandongosuchus fuyuanensis* (ZMNH M8770) in left lateral view. Scales = 1 cm in A and C and 5 cm in B.

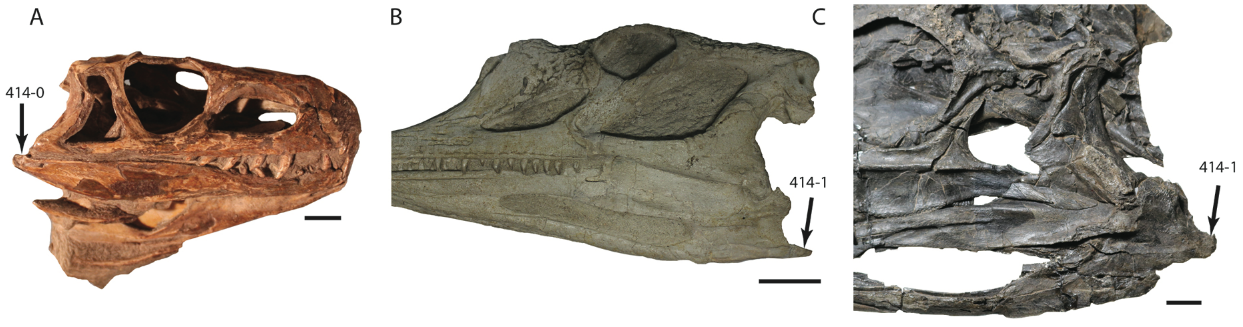

Figure S2. Comparisons of retroarticular process length and orientation among Archosauriformes. A. Holotype skull of *Euparkeria capensis* (SAM K 5867) in right lateral

view; B. Skull of *Mystriosuchus planirostris* (SMNS 13007) in left lateral view; C. Posterior one-third of holotype skull of *Diandongosuchus fuyuanensis* (ZMNH M8770) in dorsolateral view. Scales = 1 cm in A and C and 5 cm in B.

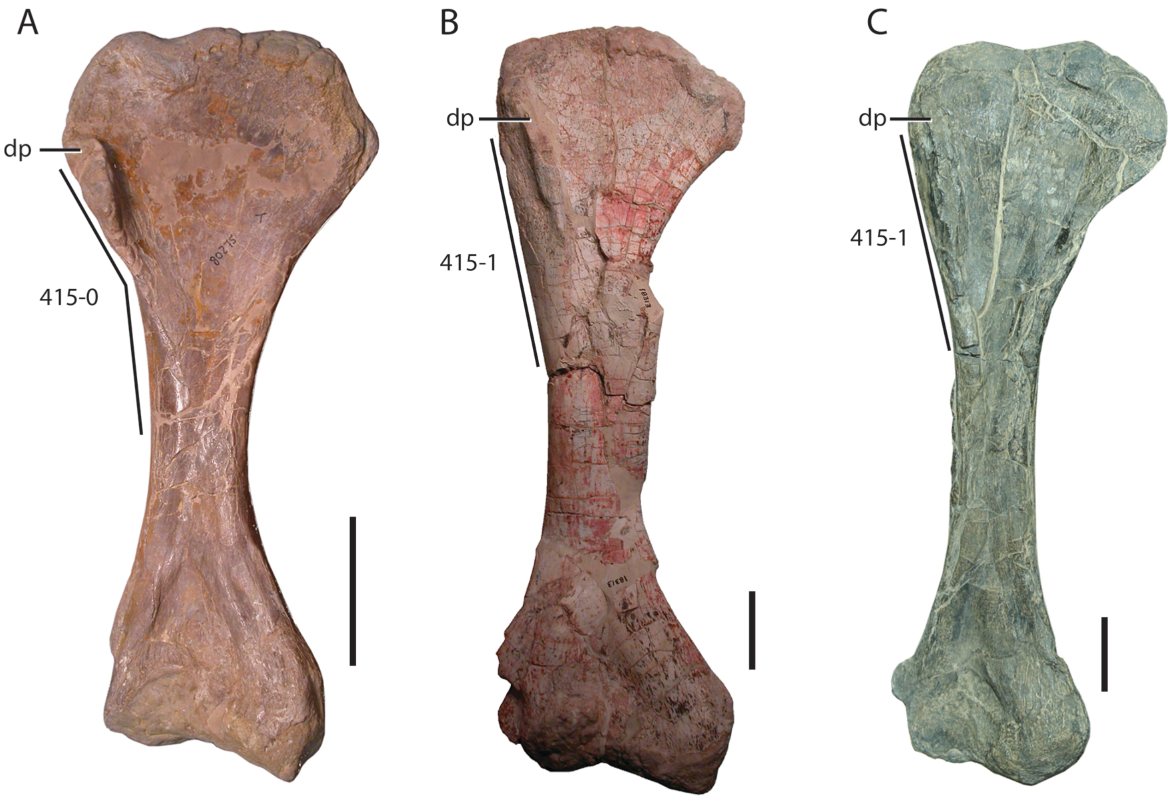

Figure S3. Comparisons of humeral morphology among Archosauriformes. A. Right humerus of *Batrachotomus kupferzellensis* (SMNS 80275) in anterior view; B. Left humerus of *Smilosuchus gregorii* (USNM 18313) in anterior view; C. Left humerus (surrounding elements erased) of holotype of *Diandongosuchus fuyuanensis* (ZMNH M8770) in anterior view. Scales = 5 cm in A and B and 1 cm in C.

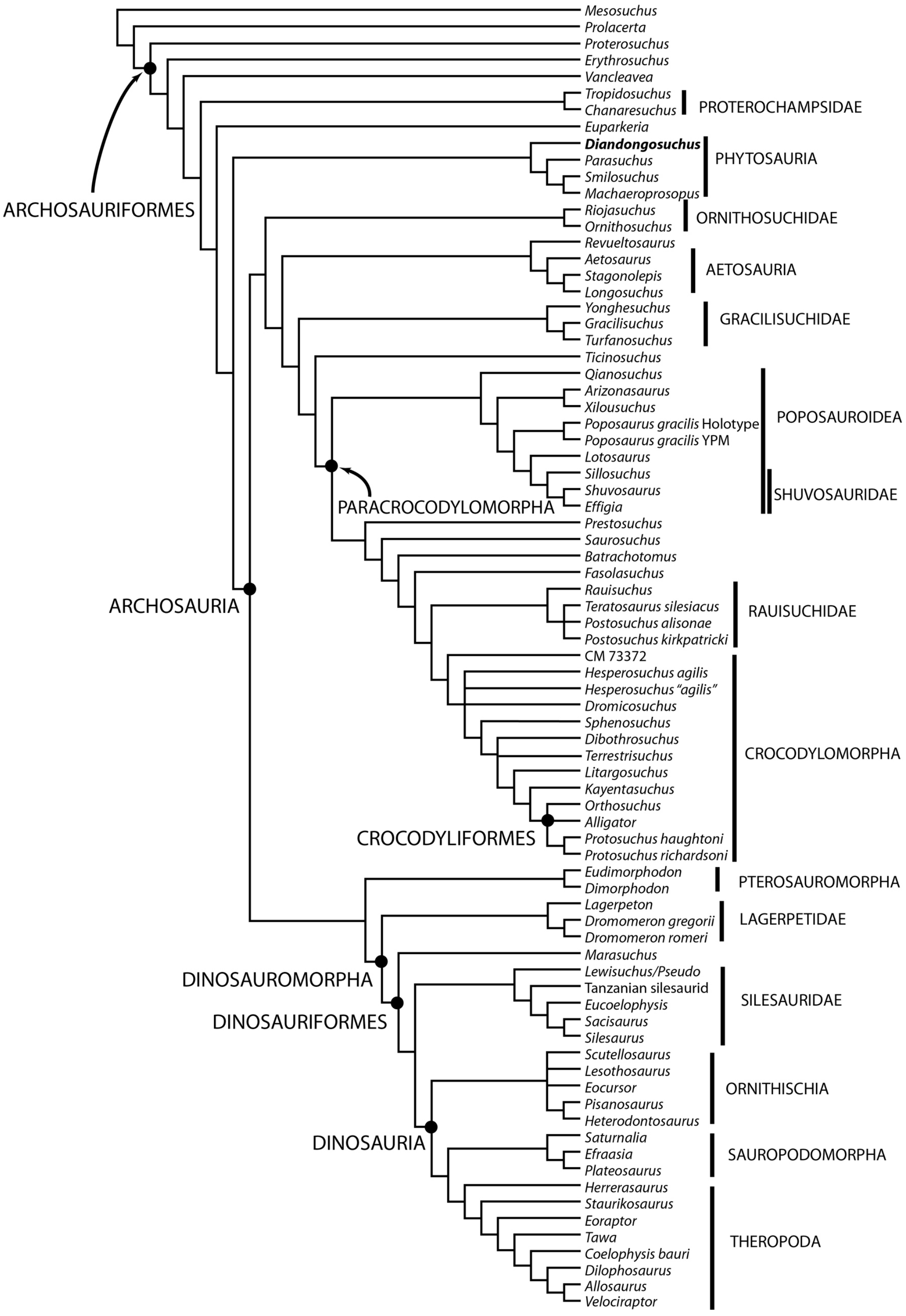

Figure S4. Phylogenetic relationships among Archosauriformes. This analysis was based on the matrix of Nesbitt[1]. *Diandongosuchus* is recovered as the basalmost phytosaur. Phytosauria is recovered here as the sister taxon to Archosauria.

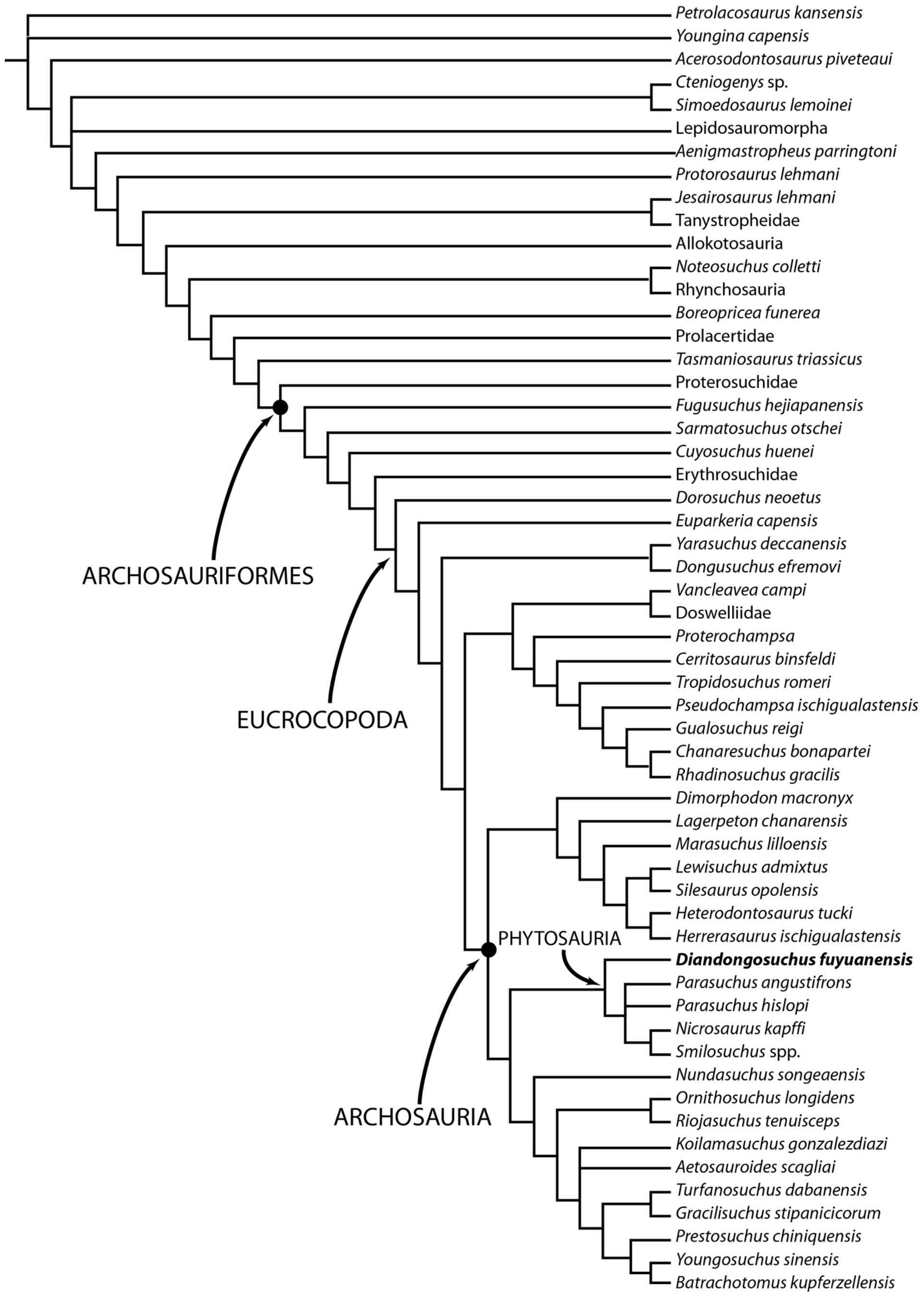

Figure S5. Phylogenetic relationships among Archosauromorpha. This analysis was based on the matrix of Ezcurra[12]. *Diandongosuchus* is recovered as the basalmost phytosaur. Phytosauria is recovered here as the earliest-branching member of Pseudosuchia within Archosauria.

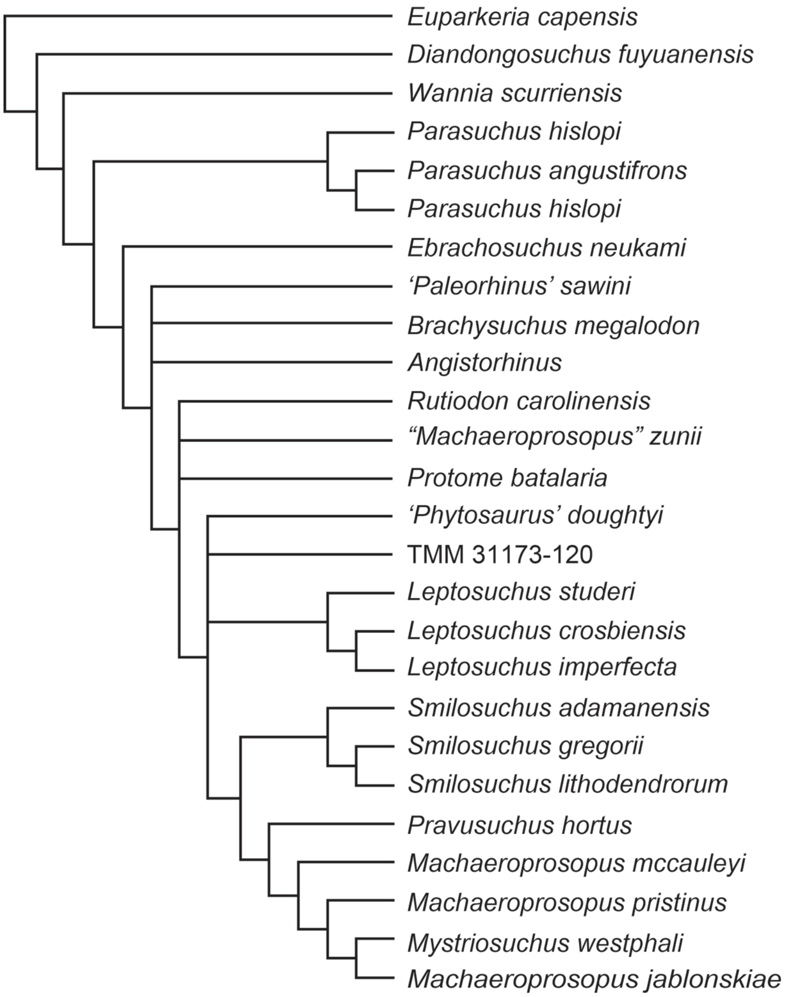

Figure S6. Phylogenetic relationships among Phytosauria. *Diandongosuchus* is recovered as the basalmost phytosaur.

IV. Support for Phytosauria outside of Archosaurs

As recovered by Nesbitt[1], we continue to find Phytosauria outside of Archosauria (Fig. S4, though this is contrary to our results using the matrix by Ezcurra[12]; Fig. S5). In his analysis, Nesbitt[1] provided the following character support for this topology that we review here with respect to *Diandongosuchus*.

Character 32: Palatal processes of maxillae do not meet on midline. This cannot currently be assessed for *Diandongosuchus* because of the compressed preservation of the holotype skull.

Character 137: Maxilla lacks antorbital fossa on dorsal edge of posteroventral process. *Diandongosuchus* does have an antorbital fossa on the dorsal edge of the posteroventral process of the maxilla.

Character 118: Very shallow lagenar/cochlear recess. This cannot currently be assessed for *Diandongosuchus* because of the compressed preservation of the holotype skull.

Character 122: The external foramen for the abducens nerve (cranial nerve VI) lies at the border between the prootic and the parabasisphenoid in phytosaurs and other non-archosaurian archosauromorphs. This cannot currently be assessed for *Diandongosuchus* because of the compressed preservation of the holotype skull.

Character 225: Posteroventral portion of the coracoid of phytosaurs is thin and lacks a ‘‘swollen’’ tuber. This cannot be seen in *Diandongosuchus* because the left coracoid has breakage and it is hidden in the right coracoid.

Character 222: Phytosaurs lack a coracoid with a postglenoid process. *Diandongosuchus* also lacks a coracoid with a postglenoid process.

Character 237: Phytosaurs, like *Euparkeria*, lack a distinct lateral tuber (=radial tuber) on the proximal portion of the ulna. *Diandongosuchus* also lacks a distinct lateral tuber on the proximal portion of the ulna.

Character 245: In phytosaurs, *Euparkeria*, and *Proterosuchus*, the longest metacarpal is longer than half the length of the longest metatarsal. The longest metacarpal is also longer than half the length of the longest metatarsal in *Diandongosuchus*.

Character 300. The proximal portions of the femora in phytosaurs lack a distinct anteromedial tuber. This is not visible in *Diandongosuchus* because only the posterior surfaces of the femora are exposed in the holotype specimen.

Character 353: Phytosaurs and other non-archosaurian archosauriforms lack a distal tarsal 4 with a distinct, proximally raised region on the posterior portion. This is not visible in *Diandongosuchus* because of preservation.

Character 366: Tibial facet of the astragalus not divided into posteromedial and anterolateral basins. This is not visible in *Diandongosuchus* because of preservation.

Character 377: Calcaneal tuber orientation ~45 degrees posteriorly relative to the transverse plane. This is not visible in *Diandongosuchus* because of preservation.

V. Supplemental References

1 Nesbitt, S. J. The early evolution of archosaurs: relationships and the origin of major clades. *Bulletin of the American Museum of Natural History* **352**, 1-292 (2011).

2 Benton, M. J. & Walker, A. D. *Erpetosuchus*, a crocodile-like basal archosaur from the Late Triassic of Elgin, Scotland. *Zoological Journal of the Linnean Society* **136**, 25-47 (2002).

3 Clark, J. M., Sues, H.-D. & Berman, D. S. A new specimen of *Hesperosuchus agilis* from the Upper Triassic of New Mexico and the interrelationships of basal crocodylomorph archosaurs. *Journal of Vertebrate Paleontology* **20**, 683-704 (2000).

4 Clark, J. M., Xu, X., Forster, C. A. & Wang, Y. A Middle Jurassic 'sphenosuchian' from China and the origin of the crocodylian skull. *Nature* **430**, 1021-1024 (2004).

5 Olsen, P. E., Sues, H.-D. & Norell, M. A. First record of *Erpetosuchus* (Reptilia: Archosauria) from the Late Triassic of North America. *Journal of Vertebrate Paleontology* **20**, 633-636 (2000).

6 Parrish, J. M. Phylogeny of the Crocodylotarsi, with reference to archosaurian and crurotarsan monophyly. *Journal of Vertebrate Paleontology* **13**, 287-308 (1993).

7 Sues, H.-D., Olsen, P. E., Carter, J. G. & Scott, D. M. A new crocodylomorph archosaur from the Upper Triassic of North Carolina. *Journal of Vertebrate Paleontology* **23**, 329-343 (2003).

8 Li, C., Wu, X.-C., Zhao, L.-J., Sato, T. & Wang, L.-T. A new archosaur (Diapsida, Archosauriformes) from the marine Triassic of China. *Journal of Vertebrate Paleontology* **32**, 1064-1081, doi:10.1080/02724634.2012.694383 (2012).

9 Butler, R. J. *et al.* New clade of enigmatic early archosaurs yields insights into early pseudosuchian phylogeny and the biogeography of the archosaur radiation. *BMC Evolutionary Biology* **14**, 1-16, doi:10.1186/1471-2148-14-128 (2014).

10 Chatterjee, S. A primitive parasuchid (Phytosaur) reptile from the Upper Triassic Maleri Formation of India. *Palaentology* **21**, 83-127 (1978).

11 Ewer, R. F. The anatomy of the thecodont reptile *Euparkeria capensis* Broom. *Philosophical Transactions of the Royal Society of London, Series B* **248**, 379-435 (1965).

12 Ezcurra, M. D. The phylogenetic relationships of basal archosauromorphs, with an emphasis on the systematics of proterosuchian archosauriforms. *PeerJ* **4**, e1778; DOI 1710.7717/peerj.1778 (2016).

13 Stocker, M. R. & Butler, R. J. in *Anatomy, Phylogeny and Palaeobiology of Early Archosaurs and their Kin Geological Society, London, Special Publications* (eds S. J. Nesbitt, J. B. Desojo, & R. B. Irmis) 91-117 (The Geological Society of London, 2013).

14 Kammerer, C. F., Butler, R. J., Bandyopadhyay, S. & Stocker, M. R. Relationships of the Indian phytosaur *Parasuchus hislopi* Lydekker, 1885. *Papers in Palaeontology*, 1-23, doi:10.1002/spp2.1022 (2015).
